# Effectiveness of electrocardiogram interpretation education program using mixed learning methods and webpage

**DOI:** 10.1186/s12909-024-05960-8

**Published:** 2024-09-27

**Authors:** Sunhee Lee, hyo jeong Kim, Young Choi, ji yeung Kim, ji sun Shin

**Affiliations:** 1 Department of Nursing, Seoul St. Mary’s Hospital, The Catholic University of Korea, Seoul, Republic of Korea; 2 Division of Cardiology, Department of Internal Medicine, Seoul St. Mary’s Hospital, College of Medicine, The Catholic University of Korea, Seoul, Republic of Korea

**Keywords:** Education, nursing, Electrocardiogram, Nurses, Program evaluation

## Abstract

**Aim:**

This study was conducted to develop an electrocardiogram education program that incorporates an HTML webpage and blended learning methods to enhance electrocardiogram interpretation skills. Through continual and efficient education, the program aims to assist nurses in providing appropriate care and treatment to patients.

**Design:**

Pre-post design study.

**Methods:**

We developed an electrocardiogram interpretation HTML webpage based on an electrocardiogram interpretation algorithm and implemented an 18-week (2023.5.15 ~ 2023.9.22) electrocardiogram education program, which included daily 5-minute training sessions. Twenty-seven ward nurses were provided with the URL (https://ecgweb.github.io/ECGwebEN) to the electrocardiogram interpretation HTML webpage and shared one electrocardiogram case daily for self-interpretation. Electrocardiogram interpretation performance and confidence were evaluated through questionnaires at three phases: before the program, after 6 weeks of basic electrocardiogram and arrhythmia education, and after 12 weeks of application of the electrocardiogram interpretation HTML webpage and case-based lecture education. The statistical tests used were repeated-measures ANOVA or the Wilcoxon signed-rank test.

**Results:**

The average score for electrocardiogram interpretation performance before the electrocardiogram education program was 11.89(SD = 3.50), after 6 weeks of basic electrocardiogram and arrhythmia education it was 14.15(SD = 3.68), and after 12 weeks of application of the electrocardiogram interpretation HTML webpage and case-based lecture education, it was 15.56(SD = 3.04). This shows that electrocardiogram interpretation performance significantly improved over time (*p* < .001). Additionally, post-hoc analysis revealed significant differences in electrocardiogram interpretation performance at each stage, i.e., before, during, and after the application of an electrocardiogram education program. Furthermore, the electrocardiogram interpretation confidence questionnaire score (pre-Median 18, IQR = 5; post-Median 23, IQR = 3) was improved significantly after the completion of the 18-week education program (*p* < .001).

**Conclusions:**

Based on the results of this study, we believe that an electrocardiogram education program using HTML webpage, and a blended teaching method would be very beneficial for maintaining and improving electrocardiogram interpretation skills of clinical nurses. Such a program can help nurses interpret electrocardiograms more effectively and assist them in making important decisions in patient care.

## Introduction

Early detection of arrhythmias is crucial for prompt defibrillation in cases of sudden ventricular tachycardia, which increases survival rates [1, 2]. It also helps identify inpatients at risk of cardiac arrest, recognizing deteriorated states that can lead to life-threatening sustained arrhythmias, thus allowing for preventive or mitigating treatment [3]. Moreover, electrocardiogram monitoring facilitates the diagnosis of non-life-threatening arrhythmias or symptom causes, enabling appropriate management, including medication [4]. Early and proper detection of and response to arrhythmias can prevent serious health issues.

Therefore, the swift and accurate interpretation of electrocardiograms by healthcare professionals is essential for promoting positive patient outcomes [5], especially for nurses in wards using electrocardiogram tele monitoring, where electrocardiogram interpretation skills are indispensable. However, studies have reported a lack of knowledge among nurses regarding electrocardiogram monitoring [6–8], highlighting the need for continual education and training to enhance their electrocardiogram interpretation skills [9].

Educational interventions involving electrocardiogram monitoring for nurses have been shown to increase their knowledge of electrocardiograms [10, 11], and education in skilled electrocardiogram interpretation is essential under pressure to detect and care for life-threatening arrhythmias. However, due to busy work schedules and numerous nursing skills and protocols for learning, dedicating ample time for education to enhancing electrocardiogram interpretation skills is challenging. Furthermore, despite various approaches to electrocardiogram interpretation, an optimal teaching method has not yet been established [12, 13].

Thus, an effective electrocardiogram education program is needed to improve basic electrocardiogram interpretation skills and the ability to interpret electrocardiograms swiftly and accurately.

This study aimed to develop an easily accessible, well-designed, and effectively usable electrocardiogram interpretation HTML webpage, applying it to mixed learning methods, including case-based learning [14] and lecture-style electrocardiogram learning, using short periods before training. The goal of this study to develop an efficient electrocardiogram education program for nurses and assess its effectiveness, enabling nurses to interpret electrocardiograms quickly and accurately, and providing appropriate nursing and treatment based on electrocardiogram changes to patients.

This study was conducted to develop an electrocardiogram education program that incorporates an HTML webpage and blended learning methods to enhance electrocardiogram interpretation skills. Through continual and efficient education, the program aims to assist nurses in providing appropriate care and treatment to patients. The specific objectives of this study are as follows:

1. To develop an electrocardiogram education program that applies mixed learning methods, including the electrocardiogram interpretation HTML webpage.
2. To compare electrocardiogram interpretation performance abilities before, during, and after the application of the electrocardiogram education program.
3. To compare confidence in electrocardiogram interpretation performance before and after the application of the electrocardiogram education program.

## Background

Electrocardiogram monitoring serves as a crucial tool for observing various electrical changes in the heart, such as arrhythmias, myocardial ischemia, and QT interval prolongation. This technology plays a vital role in the diagnosis and treatment of patients with existing cardiac conditions or those at risk of developing such conditions [15]. Continuous electrocardiogram monitoring, which is widely used in acute care settings today, enables the ongoing tracking of a patient’s cardiac status, allowing for immediate response to any arising issues.

Nurses working in cardiology departments that implement electrocardiogram telemonitoring bear significant responsibility for the care of patients undergoing electrocardiogram monitoring. Their duties include the accurate placement of electrodes, appropriate setting of alarm parameters, and making clinical decisions based on the information obtained from the monitors. The quality of patient care and outcomes is directly influenced by these tasks, underscoring the importance of nurses being well-versed in electrocardiogram interpretation.

However, studies indicate that many nurses lack the specialized knowledge required for electrocardiogram monitoring [6, 7, 11, 16], which could negatively impact patient safety and quality of care. This highlights the necessity for targeted electrocardiogram interpretation training for nurses to ensure that they possess the expertise needed to effectively contribute to patient care in settings where electrocardiogram monitoring is a critical component of clinical practice.

Recent studies have demonstrated the effectiveness of electrocardiogram interpretation training targeted at nursing students. One study revealed that a 4-week web-based electrocardiogram education program significantly improved nursing students’ ability to interpret an electrocardiogram [17]. Another study showed that nursing students who were taught using cardiac rhythm identification for simple people with a flipped classroom approach outperformed a control group receiving traditional lecture-based education in terms of electrocardiogram test scores, self-directed learning motivation, and understanding of the educational content [18].

A study comparing near-peer teaching and electronic learning among medical students revealed that those who received near-peer teaching had higher final electrocardiogram exam scores, although both educational methods improved students’ confidence [19]. These results suggest that various approaches to electrocardiogram education can be effective. However, these studies were limited to undergraduate students, and it is uncertain whether the knowledge gained from electrocardiogram interpretation training is retained in clinical practice.

The methods applied in electrocardiogram interpretation education included self-directed learning, workshop-based education, lecture-based education, web-based learning, and user-generated video sharing, with the majority being lecture-based. Web-based packages have received attention in recent years for their accessibility but have not become effective educational methods due to low completion rates and the importance of interaction during education [16]. Given these challenges, blended learning has emerged as a promising approach. It combines various information delivery methods, educational models, and both face-to-face and online learning, allowing for the design of educational programs that can include online education, in-person teaching, collaborative learning, team-based learning, problem-based and case-based learning, simulations, and online group work. This flexibility offers a way to overcome the limitations of traditional educational methods and improve the effectiveness of electrocardiogram interpretation training [20, 21].

Research on blended learning methods applied to electrocardiogram education for medical students has shown that preclass video viewing through flipped classroom design and team-based learning effectively improved electrocardiogram test scores and confidence. This was attributed to students’ preclass video viewing, which helped them understand basic concepts, and focus on solving actual problems through team-based learning during class time [21]. Another study compared the effects of standalone lectures and blended learning, which used lectures and web applications for medical students, finding that the blended learning group had better electrocardiogram interpretation skills, improved confidence, and better retention of electrocardiogram interpretation skills [22]. A study that applied traditional face-to-face education, online education, and a blended approach showed that participants who received blended education had significantly higher electrocardiogram test scores [23].

These findings suggest that blended learning methods can contribute to enhancing actual competencies and confidence in electrocardiogram education, beyond simple knowledge transmission. The combination of online materials and in-person teaching allows learners to study at their own pace, leading to a deeper understanding and practice. The proposed electrocardiogram education program for nurses in this study aimed to maximize learning effects by combining lecture-based education with electrocardiogram case studies and using an electrocardiogram interpretation webpage for automatic feedback. This approach helps nurses enhance their knowledge of electrocardiogram interpretation and develop practical skills needed in the clinical setting. Additionally, the use of web-based learning materials reduces constraints on learners’ time and location, and real-time feedback enhances learning efficiency. This study aimed to utilize this blended learning approach to enable nurses to effectively acquire and maintain electrocardiogram interpretation skills.

## Methods

### Design

This study involves the development of the electrocardiogram interpretation HTML webpage and an electrocardiogram education program that applies mixed learning methods. The effectiveness of the program was assessed by comparing the electrocardiogram interpretation performance abilities and confidence levels of nurses before, during, and after the program’s application, using a prepost design study.

### Participants

The study recruited nurses working in a cardiac unit of a tertiary hospital in Seoul, which implements electrocardiogram telemonitoring. The participants were nurses who understood the purpose of the study and who voluntarily agreed to participate. Nurses who did not complete the 18-week program were excluded. Initially, 28 participants were involved, with one excluded for not completing the program, resulting in 27 participants. The sample size was calculated using the G-Power program, with a significance level of 0.05, effect size of 0.5, and power of 0.80, using the Wilcoxon signed-rank test. Accordingly, 28 participants were needed for the study.

### Tools

#### General characteristics

Data including age, years of work experience, previous electrocardiogram educational experience, and the perceived importance of electrocardiogram interpretation skills for nurses were collected to understand the participant profile.

#### Electrocardiogram interpretation performance ability

This was measured using electrocardiogram questions and image files from the web-based electrocardiogram learning program developed by American Medical Training and Simulation [24]. The assessment items were drawn in certain proportions from six sessions of the website’s electrocardiogram reference guide (Atria, Conduction, Atrioventricular Junction, Pacemaker, Idioventricular, Ventricle). A total of 20 questions were used, and for the first, second, and third evaluations, the electrocardiogram image files were kept the same, but the positions of the questions and their answers were changed for each evaluation. Additionally, electrocardiogram image files included in the evaluation questions were excluded during case education. To enhance the discriminative power of the test, it was structured in a multiple-choice format with seven options. Correct answers were scored 1 point, and incorrect answers were scored 0 points, with the total score calculated accordingly. The total score ranged from 0 to 20 points, with higher scores indicating better electrocardiogram interpretation performance ability.

#### The performance of confidence in electrocardiogram interpretation

Confidence was measured using a scale based on an electrocardiogram textbook [25] with six items: ‘I can measure heart rate’, ‘I can distinguish whether the rhythm is regular or irregular’, ‘I can differentiate P waves, QRS complexes, and T waves’, ‘I can distinguish the relationship between P waves and QRS complexes’, ‘I can measure QRS intervals’, and ‘I can interpret arrhythmia’. These items were scored on a scale from ‘not confident at all’ (1 point) to ‘very confident’ (5 points), with the total score calculated accordingly. The total score ranged from 6 to 30 points, where higher scores indicate greater confidence in electrocardiogram interpretation performance.

### Research procedure and data collection method

#### Development of an electrocardiogram interpretation HTML webpage and educational program

**Fig. 1:**
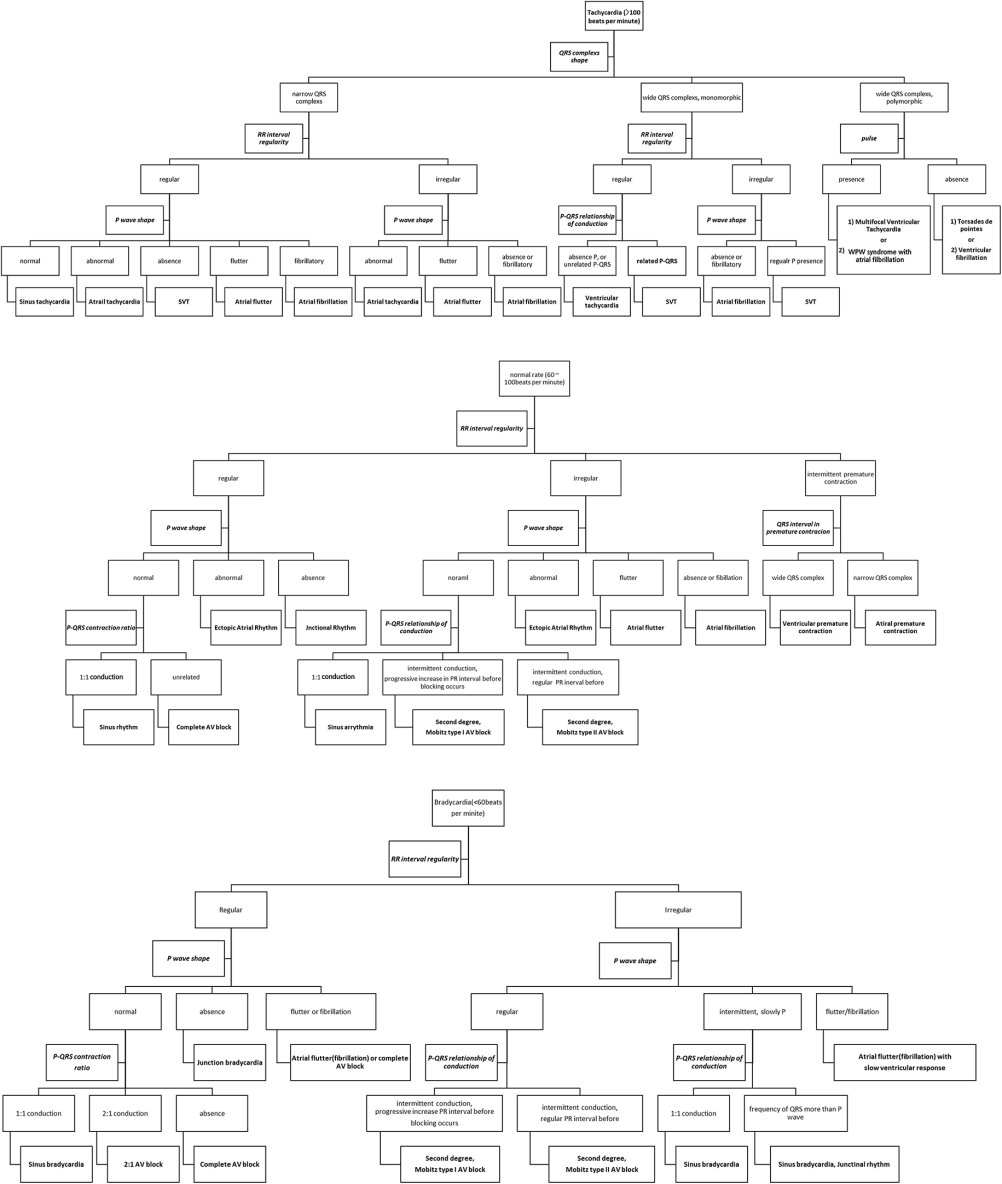
Electrocardiogram interpretation algorithm

The researcher, in collaboration with a cardiologist specializing in arrhythmia from the Department of Cardiology, developed an algorithm based on the interpretation of arrhythmias from an electrocardiogram textbook [26], focusing on heart rate, rhythm, the shape of P waves, and the relationship between P waves and QRS complexes. This algorithm was then used to create the electrocardiogram interpretation HTML webpage (Insert Fig. 1 here).

The electrocardiogram education program was developed by a researcher who has over 20 years of experience in cardiac intensive care units, arrhythmia laboratories, cardiology wards, and nursing education departments. The content was reviewed by a cardiologist specializing in arrhythmia.

#### Application of the electrocardiogram education program

The program included daily 5-minute lectures before each shift, covering basic electrocardiogram over two weeks, arrhythmias over four weeks, and a total of 12 weeks of training on 122 cases, as well as daily interpretation of one case from a total of 84 cases over 18 weeks (2023.5.15 ~ 2023.9.22) using the electrocardiogram interpretation HTML Webpage (Insert Table 1 here).

**Table 1:** ECG Education Program

Duration (Weeks)	Content	Method
2	Basic ECG Education - Electrical flow of the heart - Basic ECG - Fundamentals of ECG interpretation	Sharing educational materials and 5-minute education before work during weekdays
4	Arrhythmia Education - Bradycardia - Tachycardia - Supraventricular arrhythmias - Junctional arrhythmias - Ventricular arrhythmias	Sharing educational materials and 5-minute education before work during weekdays
12	1. Application of ECG interpretation HTML webpage 2. Case-based ECG interpretation education	1. Daily delivery of one ECG case via messenger, over 80 cases, with feedback on screenshots the next day. 2. 5-minute education before work during weekdays

**Fig. 2:**
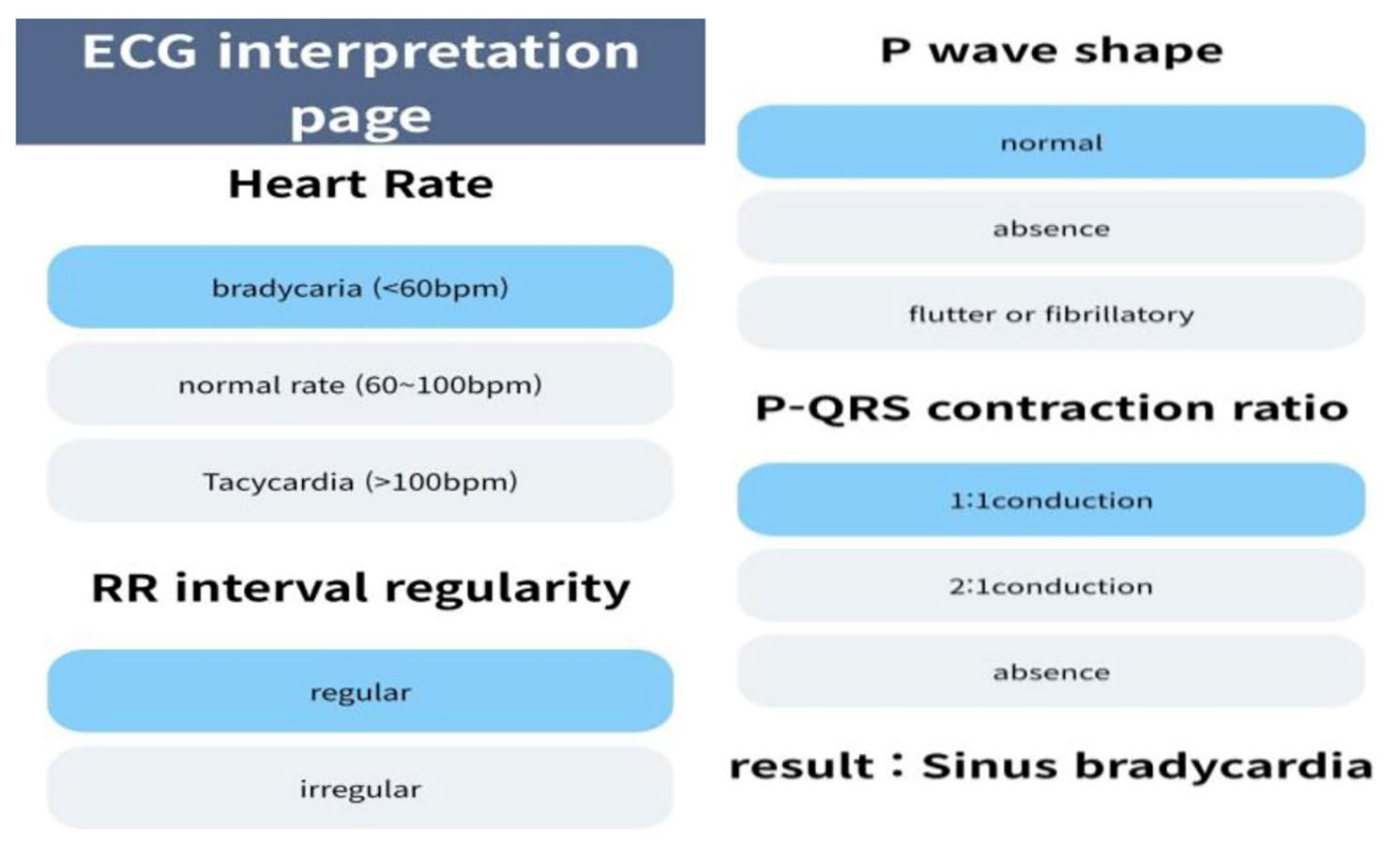
Screenshot of the electrocardiogram interpretation page

The webpage URL (https://ecgweb.github.io/ECGwebEN) was shared with all participants via messenger, and the researcher sent one case electrocardiogram daily via messenger. Participants independently interpreted the arrhythmias and shared the results the next day. The use of an electrocardiogram interpretation HTML webpage allows for the automatic generation of interpretation results when a user sequentially selects the heart rate, RR interval regularity, P wave shape, and P-QRS relationship (Insert Fig. 2 here).

The 18-week electrocardiogram lecture series consisted of daily 5-minute training sessions held before each shift, these sessions were conducted by four coresearcher nurses with over 10 years of experience in cardiac intensive care and cardiology wards. These nurses developed the training schedule and conducted the sessions based on the educational materials.

#### Electrocardiogram interpretation performance and confidence survey

Before the implementation of the electrocardiogram education program, the first test for electrocardiogram interpretation performance and a survey on confidence in performance were conducted. After two weeks of basic electrocardiogram and four weeks of arrhythmia education, the second test for electrocardiogram interpretation performance was administered. Following the application of the electrocardiogram Interpretation HTML Webpage and 12 weeks of lecture-style case study education, the third test for electrocardiogram interpretation performance and a survey on confidence in performance were conducted.

### Data analysis

The collected data were analyzed using Python 3.8. The normality of the electrocardiogram interpretation performance data before, during, and after the implementation of the electrocardiogram education program was tested using the Shapiro-Wilk test. The results showed Shapiro-Wilk significance probabilities of 0.466, 0.392, and 0.063, indicating a normal distribution. Therefore, the differences in electrocardiogram interpretation performance before, during, and after the application of the electrocardiogram education program were analyzed using repeated-measures ANOVA, with Tukey’s HSD as the post-hoc test. For the data on confidence before and after the application of the electrocardiogram education program, the postimplementation data did not follow a normal distribution, so the Wilcoxon signed-rank test was used for analysis.

### Ethical considerations

The study was approved by the Institutional Review Board (KC23QISI0247) and was conducted following the receipt of informed consent from the participants. Participants were informed that they could withdraw consent at any time.

## Results

### General characteristics

The average age of the participants was 27.56 ± 3.34 years, and the average work experience was 3.56 ± 3.39 years, with 13 participants (48.1%) having 2–3 years of experience. Ten nurses (37.0%) had previous electrocardiogram education experience. Regarding the importance of electrocardiogram interpretation skills for nurses, 16 participants (59.3%) answered that it is ‘important’, and 10 participants (37.0%) said it is ‘very important’, with 96.3% considering it at least ‘important’.

### Differences in electrocardiogram interpretation performance before, during, and after the electrocardiogram education program

Before electrocardiogram education, the average score for electrocardiogram interpretation performance was 11.89(SD = 3.50) out of 20. After basic electrocardiogram and arrhythmia education, the average score was 14.15(SD = 3.68), and after using the electrocardiogram Interpretation HTML Program and case study education, the average score was 15.56(SD = 3.04). There was a significant difference in electrocardiogram interpretation performance over time (F = 7.91, *p* < .001), with post hoc analysis showing significant improvements after both basic electrocardiogram and arrhythmia education (*p* < .001) and after using the electrocardiogram Interpretation HTML Program and case study education *(p* < .001) compared with before education. The average score after using the electrocardiogram Interpretation HTML Program and case study education was significantly greater than that after basic electrocardiogram and arrhythmia education (*p* = .017) (Insert Table 2 here).

**Table 2:** ECG interpretation performance before and after applying the ECG Education Program. *N* = 27

	Mean ± SD	F	*p*	Tukey
Pre^a^	11.89 ± 3.50			
During^b^	14.15 ± 3.68	7.91	< 0.001	a < b < c
Post^c^	15.56 ± 3.04			

An examination of the six detailed items, revealed that the average scores for ‘measuring heart rate’ increased from 3.48(SD = 0.98) before the application of the program to 4.15(SD = 0.60) after its application. The average score for ‘distinguishing whether the rhythm is regular or irregular’ increased from 3.63(SD = 0.84) to 4.00(SD = 0.56). The scores for ‘identifying P waves, QRS complexes, and T waves’ increased from 3.26(SD = 0.76) to 3.85(SD = 0.60). For ‘differentiating the relationship between P waves and QRS complexes’, the score increased from 2.93(SD = 0.73) to 3.59(SD = 0.64). The average score for ‘measuring QRS intervals’ increased from 2.78(SD = 0.64) to 3.70(SD = 0.61), and for ‘interpreting arrhythmias’, it improved from 2.44(SD = 0.80) to 3.22(SD = 0.42) after the application of the electrocardiogram program. Thus, scores increased for all items following the implementation of the electrocardiogram program.

The item ‘interpreting arrhythmias’, which had the lowest average score before the application of.

the electrocardiogram program, remained the lowest even after the application. Before the electrocardiogram program was applied, the highest average score was for ‘distinguishing whether the rhythm is regular or irregular’, and after applying the electrocardiogram program, the highest average score was for ‘measuring heart rate.’

### Differences in confidence in electrocardiogram interpretation performance before and after the electrocardiogram education program

The questionnaire score for electrocardiogram interpretation confidence was 18.52(SD = 3.38) out of 30 before the application of the program and 22.52(SD = 2.28) after the completion of the 18-week electrocardiogram education program. The increase in the confidence scores before (Median 18 IQR = 5) and after (Median 23 IQR = 3) the electrocardiogram education program was statistically significant (*p* < .001) (Insert Table 3 here).

**Table 3:** Confidence in ECG interpretation performance before and after applying the ECG Education Program. *N* = 27

	Mean ± SD	Median (IQR)	*p*
Pre	18.51 ± 3.38	18(5)	< 0.001
Post	22.52 ± 2.28	23(5)	

*Wilcoxon signed-rank test

## Discussion

This study aimed to develop an electrocardiogram interpretation HTML webpage and apply a blended learning method in an electrocardiogram education program for nurses to assess its effectiveness.

This study investigated the experience of ward nurses with electrocardiogram education and their perceptions of electrocardiogram interpretation skills. Only 37.0% of ward nurses had received electrocardiogram training, whereas a much greater percentage, 96.3%, recognized the importance of electrocardiogram interpretation skills. In comparison, a study [27] involving on ICU nurses revealed that 69.2% had electrocardiogram training experience, which is significantly greater than the percentage of ward nurses included in this study. This difference might be due to ICU nurses having an average clinical experience of 5.18 years, compared to the 3.56 years of experience of the ward nurses in this study, suggesting that nurses with shorter career lengths may have fewer opportunities for electrocardiogram education. Additionally, ICU nurses likely have more opportunities for electrocardiogram training due to the implementation of bedside electrocardiogram monitoring in the ICU. The high recognition of the importance of electrocardiogram interpretation skills among nurses in this study could be attributed to the ward’s focus on caring for cardiology patients and the application of electrocardiogram telemonitoring, indicating that participants viewed electrocardiogram interpretation skills as essential in their work environment. This perception may have influenced their motivation to participate in the electrocardiogram education program proposed by this study.

A blended learning approach was used to provide lecture-style education on basic electrocardiograms, arrhythmias, and electrocardiogram case studies. This education was conducted for 5 min before the start of each work shift every weekday. Additionally, nurses were provided with the electrocardiogram interpretation HTML webpage and electrocardiogram cases via messenger, guiding them to interpret the electrocardiogram independently.

To evaluate the effectiveness of this blended learning approach in electrocardiogram education program, this study compared electrocardiogram interpretation performance and confidence in interpretation before, during, and after the program’s application. According to the study results, electrocardiogram interpretation performance improved over time, demonstrating the effectiveness of the blended learning approach in electrocardiogram education programs. These results were similar to those found in Go’s study [2], which applied a web-based electrocardiogram learning program to nursing students. However, while Go’s study focused on a one-time, 2.5-hour education session, this study provided continuous education for 5 min daily over 18 weeks, using the electrocardiogram interpretation HTML webpage for case study education, allowing for future clinical electrocardiogram interpretation assistance in a mobile web format.

This study demonstrated that continuous and brief daily education combined with digital resources is effective in improving electrocardiogram interpretation performance in a demanding medical environment.

Repeated measurements over three time points confirmed the lasting effectiveness of the blended learning method in electrocardiogram education and the effectiveness of case study education using the electrocardiogram interpretation HTML webpage based on basic electrocardiogram knowledge.

As a result of this study, confidence in electrocardiogram interpretation performance significantly increased after applying the blended learning method in the electrocardiogram education program compared to before its application. This increase in confidence is consistent with findings from a study that showed improved confidence and knowledge in electrocardiogram interpretation after education, regardless of group assignment, whether it was traditional, e-learning alone, or a combination of both [27]. However, traditional lecture-style education has limitations as not all nurses can attend, and e-learning might vary in effectiveness depending on the nurse’s internal motivation and self-directed learning style, with less opportunity for interaction with educators [27]. In contrast, the blended learning method in this study allowed all nurses to participate in 5-minute education before work and enabled interaction with educators based on real cases, providing efficient education through electrocardiogram interpretation assisted webpages.

Electrocardiogram education through various methods has increased confidence in electrocardiogram interpretation. This boost in confidence plays a crucial role in the decision-making process when treating patients with arrhythmias and other significant cardiac issues, potentially directly impacting patient care outcomes. Additionally, this confidence enhances nurses’ professional self-concept and job satisfaction, positively affecting their intention to stay in their positions in the long term [28, 29]. Therefore, the importance of continuing education and training to improve electrocardiogram interpretation skills should be further emphasized in the clinical setting.

This study detailed the effect of an electrocardiogram education program on confidence in performing electrocardiogram interpretations, showing that, despite an increase in the average score for the ‘arrhythmia interpretation’ item, which had the lowest confidence before the program’s application, it remained the lowest scoring item afterward. This suggests difficulties in understanding and integrating the various characteristics of electrocardiograms for accurate interpretation.

The electrocardiogram interpretation HTML webpage developed for this research likely included features for tracking learners’ progress and providing immediate feedback, which are vital for effective learning. The webpage component of the educational program was essential in creating a flexible and engaging learning environment, significantly enhancing the electrocardiogram interpretation skills of the nurses. Furthermore, ongoing use of this electrocardiogram interpretation HTML webpage could lead to further improvements in electrocardiogram interpretation skills in the future.

## Limitations

The application of the electrocardiogram education program in this study faced a constraint that required an educator with the capability to conduct electrocardiogram education to be included as a member of the department implementing the program. This requirement stems from the nature of the education, which needs to be conducted before work. Additionally, in the 5-minute lecture-style electrocardiogram education program, conducted before work, was somewhat limited for nurses who were not on duty, as the program specifically targeted nurses on duty. To minimize differences in educational effects resulting from this limitation, all educational materials were shared. However, such constraints could still impact the effectiveness of education.

## Conclusions

Based on the results of this study, we believe that an electrocardiogram education program using HTML webpages and a blended teaching method would be very beneficial for maintaining and improving the electrocardiogram interpretation skills of clinical nurses. Such a program can help nurses interpret an electrocardiogram more effectively and assist them in making important decisions in patient care. Additionally, complex electrocardiogram cases, such as bundle branch blocks and pacemaker rhythms, which cannot be analyzed through HTML webpages, should be included in briefcase-based training sessions before work to continue education.

## Data Availability

The dataset generated and analyzed during the current study is available in the following link: https://docs.google.com/spreadsheets/d/1eBrzTtPkLjagQeDY_a02YsbHCIPWcHVo/edit? usp=sharing&ouid=118257517890635267381&rtpof=true&sd=true.
